# The Strategic Alliance between Clinical and Molecular Science in the War against SARS-CoV-2, with the Rapid-Diagnostics Test as an Indispensable Weapon for Front Line Doctors

**DOI:** 10.3390/ijms21124446

**Published:** 2020-06-22

**Authors:** Antonio Vittorino Gaddi, Fabio Capello, Leonardo Aluigi, Pier Luigi Antignani, Annapaola Callegaro, Gavino Casu, Enrico Cipolla, Maurizio Cipolla, Lucio Cosco, Federico Culzoni, Francesco Dentali, Maria Elexpuru-Zabaleta, Tamara Y. Forbes-Hernandez, Claudia Fragiacomo, Francesca Giampieri, Agostino Gnasso, Raffaele Mancini, Maria Grazia Modena, Michele Nichelatti, Angelo Virgilio Paradiso, Pasquale Ortasi, Maria Teresa Savo, Flavio Tangianu, Sergio Tempesta, Tommaso Diego Voci, Maurizio Battino

**Affiliations:** 1 Society of Digital Health and Telemedicine, 40100 Bologna, Italy; antonio.gaddi@ehealth.study; 2 International Study Center of Society of Telemedicine and Digital Health, 40100 Bologna, Italy; fabio.capello@ehealth.study; 3 Villalba Private Hospital (GVM), 40100 Bologna, Italy; l.aluigi@libero.it; 4 Vascular Centre, Nuova Villa Claudia, 00191 Rome, Italy; antignanipl@gmail.com; 5 Department of Laboratory Medicine ASST-Papa Giovanni XXIII, 24127 Bergamo, Italy; acallegaro@asst-pg23.it; 6 Cardiology Dept. – ATS Sardegna ASSL, San Francesco Hospital, 08100 Nuoro, Italy; gavino.casu@atssardegna.it; 7 Alma Mater Studiorum Università di Bologna, 40126 Bologna, Italy; enrico.cipolla@studio.unibo.it; 8 UCCP Catanzaro Lido, ASP Catanzaro; Calabria Society of Telemedicine-Regione Calabria, 88100 Catanzaro, Italy; cipolla.maurizio54@gmail.com; 9 Infectious Disease Department., “Pugliese-Ciaccio” Hospital, 88100 Catanzaro, Italy; luciocosco@alice.it; 10 Culzoni Pharmacy, 20019 Milano, Italy; federicoculzoni@me.com; 11 Department of Clinical Medicine Insubria University Varese, 21100 Varese, Italy; francesco.dentali@uninsubria.it; 12 Dipartimento di Scienze Cliniche e Molecolari, Facoltà di Medicina, Università Politecnica delle Marche, 60131 Ancona, Italy; mariaelexpuru@gmail.com; 13 Nutrition and Food Science Group, department of Analytical and Food Chemistry, CITACA, CACTI, University of Vigo-Vigo Campus, 32004 Vigo, Spain; tforbes@uvigo.es (T.Y.F.-H.); f.giampieri@univpm.it (F.G.); 14 Moncucco Clinic, 6900 Lugano, Switzerland; cdisme@bluewin.ch; 15 Dipartimento di Scienze Cliniche Specialistiche e Odontostomatologiche, Università Politecnica delle Marche, Via Ranieri 65, 60130 Ancona, Italy; 16 College of Food Science and Technology, Northwest University, Xi’an 710069, China; 17 Department of Applied Medical Science, Magna Graecia University, 88100 Catanzaro, Italy; gnasso@unicz.it; 18 Endocrinology and Diabetes Units, ASP, 88100 Catanzaro, Italy; raffaelemancini56@gmail.com; 19 Surgical, Medical, Dental and Morphological Science Department with Transplantology, Oncological and Rigenerative Address, Modena e Reggio Emilia University, 41125 AOU Modena, Italy; mariagrazia.modena@unimore.it; 20 Service of Statistics, Fondazione Malattie del Sangue Niguarda Hospital, 20162 Milano, Italy; michele.nichelatti@ospedaleniguarda.it; 21 IRCCS-Istituto Tumori “Giovanni Paolo II”, 70124 Bari, Italy; a.paradiso@oncologico.bari.it; 22 Primary health care Department, ASL Area Vasta Romagna, National Medical Interdisciplinary Primary health care Ravenna-Forlì-Cesena, Society of Digital Health and Telemedicine, Emilia Romagna, 48121 Ravenna, Italy; pasquale.ortasi@medici.progetto-sole.it; 23 Dipartimento di Medicina Interna, University of Florence, 50134 Firenze, Italy; 24 ASST Settelaghi Varese, Medical and Surgical Department, Insubria University, 21100 Varese, Italy; flavio.tangianu@asst-settelaghi.it; 25 Technobios Prenatale Eurogenlab-Caravelli Group, Medical Genetics Laboratory, 40126 Bologna, Italy; sergio.tempesta@geneticatpe.it; 26 Associazione Interregionale Cardiologi e Specialisti Medici Ambulatoriali, ACSA, 10125 Torino, Italy; tommasodiegovoci@gmail.com; 27 International Research Center for Food Nutrition and Safety, Jiangsu University, Zhenjiang 212013, China

**Keywords:** serological assays, immunochromatographic rapid test, colloidal gold rapid test, sensitivity, specificity

## Abstract

Our work concerns the actual problem of spread of SARS- CoV-2 outbreak which requires fast and correct as possible answer. In current scenario, the need of rapid answer put away the imperative of proper methodology. We focus on the serogical immunoassay for diagnosis of Covid-19 as an important weapon not only for diagnostic purpose, but also for epidemiologic one. The right equilibrium between high speed, low cost and accuracy is obtained with easy-to-use decentralized point-of-care test as the colloidal gold-based immunochromatographic strip assay which detects IgM and IgG antibodies directed against SARS-CoV-2. As our aim is to evaluate the efficacy of Covid-19 rapid tests and of serological assays in real-life settings, we designed a research protocol aimed to establish how to use correctly these diagnostics, taking into account the different possible clinical and epidemiological scenarios.

## 1. Introduction

The COVID-19 pandemic is probably the most challenging health crisis of the modern era. As of April 2020, more than 180 countries around the world have registered at least one confirmed case of the novel coronavirus infection, with more than 2.5 million positive patients around the world [1].

As the World Health Organization’s general director pointed out, testing is crucial [2]. The detection of SARS-CoV-2, the virus that causes COVID-19, is achieved via the detection of the viral RNA by real-time polymerase chain reaction (RT-PCR) [3]. When properly collected and if the specimens collected for the analysis contains the virus, RT-PCR has high specificity and sensitivity. However, this technique has some limitations, such as the narrow number of patients that can be tested in a given time, the increased risk of exposure to a high-dose of virus for the healthcare workers collecting the sample (throat-swabbing can generate aerosol) and a considerable amount of time from the test to its response [4]. In addition, if the sample is not properly collected, which may be the case in a mass emergency screening where a single operator performs hundreds of swabs in a single shift, this may lead to a high number of false-negative results, with an overall sensitivity of about 30–40% [5,6]. Furthermore, the RT-PCR requires certified laboratories, expensive equipment, and trained technicians to operate.

Finally, a major constraint remains the cutoff value to ascertain whether a test is positive or not. Generally, when a given value is reached, the specimen tested is considered positive. However, the degradation of the probe-based fluorophore, by cross-contamination or by nonspecific amplification of background nucleic acids, forces the adoption of an arbitrary cut off, below which the medical report is given as negative. This operating procedure, outside the clinical context, can lead to evaluation errors, raising the number of false-negative results. Moreover, real-time amplification efficiency varies within and between laboratories, and a set cutoff might not be constant across runs. Therefore, it is recommended to normalize cutoff with relative or absolute quantification approaches [7].

The World Health Organization (WHO) has fostered the development of new strategies to standardize the diagnosis of COVID-19 [8]. Yet, most countries have been completely unprepared for the onslaught of the virus.

A possible alternative to RT-PCR is the assessment of the immune response of the host to the virus. However, we cannot research Covid-19 by merely translating the technologies and the know-how already in place, and that comes from the study of other not comparable diseases. In these three months since the beginning of the outbreak, no protocols or real experimental studies have been carried out. We still do not know about the immune response of the human body to SARS-CoV-2, and it is still unclear how we can evaluate the antibody production for the diagnosis of Covid-19 and how to use IgM and IgG detection in single patients and the population for epidemiological purposes. Only recently, the idea of producing easy-to-use decentralized point-of-care (POC) tests emerged and then deploying them widely [9]. POC tests are simple, easy to administer and to interpret, and they can accelerate clinical decision-making and take some of the workload off centralized test laboratories; for example, Sheridan [9] recently reported the current main commercial rapid test for SARS-CoV-2 in Nature Biotechnology.

Some are ten-min lateral flow immunoassays that detect IgM and IgG antibodies directed against SARS-CoV-2 [Xiamen AmonMed Biotechnology (Fujian, China; Technical specifications available from http://www.biotime.cn/En_Pr_index_gci_80.html), Sugentech (Daejeon, Korea; Technical specifications available from https://sugentech.com/products/products-view.php?ct=7&target=32), and Cellex]. Biotech (Guangzhou, China; Technical specifications available from https://en.wondfo.com.cn/products/?catId=637&level=1) developed a similar test called Wondfo SARS-CoV-2 antibody test. Other similar tests promoted by Jiangsu Medomics Medical Technologies (Nanjing, China) [10] and Innovita Biological Technology are now in shipping. Abbott produced the POC PCR isothermal test that delivers a positive result in five minutes and a negative result in thirteen minutes [11] approved by the Food and Drug Administration (FDA) for emergency use (EU). The FDA approved a multiplex PCR test as well that runs on the automated ARIES system and delivers a result in two hours. Snibe Diagnostic (Shenzhen, China; Technical specifications available from http://www.snibe.com/zh_en/en_products.aspx?id=65) created an automated central laboratory rapid test that runs on the MAGLUMI chemiluminescence immunoassay system. Pharmact (Berlin, Germany) deployed the POC 20-min test for detecting SARS-CoV-2 exposure through the identification of IgG and IgM antibodies. Biotech, Xiamen AmonMed Biotechnology, Sugentech, Snibe Diagnostic and Pharmact obtained the CE mark for their serological test. Cellex developed a similar one, which is now also approved for the EU by the FDA.

The available POC tests need to be studied in the current emergency setting. The WHO proposed a protocol template better to assess the effectiveness of serology testing [12]; this is also consistent with what is suggested in the updated version of “Laboratory testing for coronavirus disease (COVID-19) in suspected human cases” [8]. However, it is currently impossible to assess and compare all the different POC tests because of the lack of reliable data.

As our aim is to evaluate the efficacy of Covid-19 rapid tests and serological assays in real-life settings with a particular focus on territorial emergencies and the ongoing pandemic, we designed a research protocol aimed to establish how to use these diagnostics correctly, taking into account the different possible clinical and epidemiological scenarios.

## 2. Serological Assay for the Diagnosis of SARS-CoV-2 Infection: Background

COVID-19 is the clinical presentation of the infection of the virus SARS-CoV-2 [13]. The diagnosis is currently based on the detection of the RNA of the virus via a nucleic acid amplification test (NAAT). According to Corman et al. [14], RT-PCR has a sensitivity and specificity of about 100%, provided that the sample is taken with the proper and timely technique. The choice of the perfect samples to use for RT-PCR is still under debate. Wenling et al. [15] analyzed samples from bronchoalveolar lavage fluid, sputum, nasal swabs, fibro-bronchoscopy (brush biopsy), pharyngeal swabs, stools, and blood. RT-PCR on bronchoalveolar lavage fluid demonstrated the highest positive rates (93%), followed by sputum with 72%. Recent studies suggest the use of RT-PCR on stools because the virus can attack the bowel [16]. According to Ai et al. [17], the combined use of Computed Tomography (CT) and PCR can increase diagnostic sensitivity. However, CT often is not available in territorial settings and primary care or for screening purposes. According to El-Tholoth et al. [18], RT-PCR has some limitations for the screening and diagnosis of COVID-19, because the test is difficult to use in the community, and because of the high number of false-negatives observed.

The detection of specific IgM and IgG antibodies against SARS-CoV-2 is a possible strategy to diagnose the infection. The immune response triggered by the infection is currently under investigation. Similarly, the long-term modification of the antibodies’ levels cannot be known at the moment. The use of a fast performing, validated, a serological assay is requested by different authors, such as Xiao [19] and Hick [20], but also the National Health Commission and State Administration of Traditional Chinese Medicine suggests the use of a serological test with IgM and IgG for Covid-19 to confirm the negative results. An emblematic example came from the recent Italian experience: “Patient zero” was negative to all tests performed during the onset of the symptoms, namely the pharyngo-nasal swab for RT-PCR and the serological test [19]. These observations support the idea that two negative serological tests are necessary to rule out the infection. When a quantitative analysis is available, the following procedure should be respected: Two paired serum samples should be taken from the patient with a suspected diagnosis of COVID-19 (patients with clinical symptomology suggestive of the disease, or close contacts with patients with a known positive test for SARS-CoV-2 infection). Baseline serum should be taken as early as possible within the incubation period of contact, and convalescent serum should be taken 2–4 weeks after the last contact. Positive serum-specific IgM, or specific IgG antibody title in the recovery phase ~4 times higher than that in the acute phase, can be used as diagnostic criteria for suspected patients with negative NAAT. IgM is usually measurable 10 days after symptom onset, while IgG is detectable 12 days after the patient is symptomatic. In fact, the optimal timing for the convalescent sample needs still to be established.

When compared to NAAT, the serological test appears more suitable for screening purposes in a population. A recent study from Jin et al. [20] emphasizes the utility of the serological test in a wide cohort, also when there are few positive patients (not only when the probability pre-test is high); Bai et al. [21] used IgM to define negative and positive case in wide samples from a general population and encouraged the use of the serological test also in asymptomatic patients to confine the spread of the outbreak. This is also consistent with an interesting article by Li et al. [22] that recommends the use of serological surveys to determine the incidence of subclinical infections.

This test, however, may have some limitations. To avoid false-positives because of cross-reactivity, the antigen used for the serological test should come from properly purified SARS-CoV-2. According to Meyer et al. [23], and following the WHO’s recommendations, the right choice and the appropriate purification of antigens is essential to obtain validated serological tests. In fact, cross-reactivity to other coronaviruses can be challenging. Therefore, only the most recent tests—validated from laboratories that have isolated the virus—can be used.

Serum antibody determination methods include, among others, colloidal gold immunochromatography, ELISA (enzyme-linked immunosorbent assay), chemiluminescence immunoassay. Xiang et al. [24] recently compared ELISA to GICA (colloidal gold-immunochromatographic assay). Both have high sensitivity and specificity without a significant difference (H0/HA 0.05).

To enhance the accuracy of the diagnosis, Liu et al. [25] suggested using RT-PCR in the first days from the infection and then to use the serological test alone for the disease monitoring.

Li et al. [10] described the results from the use of a rapid serological test in 525 patients. The test had a sensitivity of 88.6% and a specificity of 90.63% for the diagnosis of SARS-CoV-2.

Immune response to a first-time infection needs time to grow, symptoms can occur while the organism is preparing to fight, and in the first days from infection, the immunoassay can be negative. Indeed, no cross-reactivity was detected in subjects with previous coronaviruses infection [26]. A similar level of precision is reported by Cassaniti et al., in a letter to the editor, where the authors do not endorse the use of the colloidal gold rapid test in emergency settings for triage of patients with suspected COVID-19 [26]. Nevertheless, this recommendation remains controversial as there are some inconsistencies within the results presented, the sampling methods, the data processing, and the patients targeted for the study. On the other hand, more recently, Long Q.X. et al. emphasized the importance of serological testing both in clinical and epidemiological settings [27].

## 3. Methods

### 3.1. The Immunochromatographic Rapid Test—GICA

A rapid test for SARS-CoV-2 is the colloidal gold-based immunochromatographic strip assay. Zhang et al. [27] demonstrated that the eukaryotic spike proteins expressed (rS1 and rS-RBD-mFc) are more appropriate than the prokaryotic nucleocapsid proteins for Covid-19 serological diagnosis. The GICA sandwich used to detect total antibodies is a powerful complement to the current standard RNA-based tests. They prepared six recombinant proteins, including three S (Spike) proteins (rS1, rS-RBD, rS-RBD-mFc) and three N (Nucleocapsid) proteins (rN, rN1, rN2). Because the S protein is a transmembrane protein, three S protein fragments were prepared using a eukaryotic expression system, while three N proteins were prepared using a prokaryotic expression system. Preliminary evaluation by indirect ELISA revealed that the three S proteins (rS1, rS-RBD, rS-RBD-mFc) were superior in performance (lower background and higher Optical Densit values) to the three N proteins. However, the antigenicity of S proteins and that of the N proteins could not be confirmed in this study, because the influence of prokaryotic expression method used to produce the N proteins is difficult to predict. They speculated that this difference is due to the relatively high sensitivity and early response to the S antigen compared to the N antigen in patients with COVID-19. Thus, the N protein’s antigenicity should be studied after expression in a eukaryotic system in future.

Zhang et al. [28] selected two recombinant proteins with the highest ELISA titers (rS1 and rS-RBD-mFc) to develop a sandwich-format GICA strip to detect total antibodies (IgM and IgG) against HCoV-19. GICA (colloidal gold immunochromatography assay), a simple and rapid serological method, is especially suitable for timely diagnosis and large-scale sample screening. The performance of the GICA strip, which was evaluated with 814 clinical samples, showed to be rapid (10 min), highly sensitive (86.89%), and specific (99.39%) by diagnosing COVID-19 in RT-PCR-negative patients who were clinically confirmed COVID-19 positive by CT. A recent study showed that the sensitivity of the total antibody (IgM and IgG) test is higher than that of the IgM or IgG test [29].

### 3.2. Vivadiag™

The VivaDiag™ kit, an example of colloidal gold rapid test, is produced by Jiangsu Medomics Medical Technologies and may be another potential candidate for reliable and rapid (15 min) testing. The test is based on a lateral flow qualitative immunoassay for the rapid determination of the presence or absence of both anti-SARS-CoV-2-IgM and anti-SARS-CoV-2-IgG in human specimens (whole blood, serum, and plasma). The test has been reported to be based on the utilization of anti-human IgG and anti-IgM against the recombinant antigen representing the receptor-binding domain of the COVID-19 spike protein (MK201027), developed and purified at Medomics. The recombinant antigen is transient transfected in cell culture and purified by protein A affinity chromatography and size-exclusion chromatography (SEC) [10]. The design of the antigen was based on the published SARS-CoV-2 sequence. The strip test is composed of nitrocellulose membrane, with mouse anti-human-IgM monoclonal antibody, mouse anti-human-IgG monoclonal antibody, and anti-rabbit-IgG immobilized in different positions (M and G lines) and the control line (C line), respectively. When testing, 10–15 µL of the specimen was added into the sample port, followed by the addition of sample dilution buffer. The blood samples move forward along the test device, which was sprayed with a mixture of AuNP-COVID-19 recombinant antigen conjugate and AuNP-rabbit-IgG. If the sample contains IgM antibodies, antibodies will bind to the virus antigen labeled with colloidal gold, then form a sandwich complex with the coated anti-human IgM monoclonal antibody at the IgM line (M line), The IgM line will appear purplish red, prompting the COVID-19 IgM antibody is positive. If the sample contains IgG antibodies, antibodies will bind to the virus antigen labeled with colloidal gold, then form a sandwich complex with the coated anti-human IgG monoclonal antibody at the IgG line. The IgG line will appear purple–red, showing the COVID-19 IgG antibody is positive. If either line IgG or IgM does not show color, the negative result will be displayed. The test device also contains a quality control line C; whether there is a test line or not, the quality control line C should display. Each test was evaluated by two operators, and a picture was taken.

#### Analysis of Preliminary Data on the Reliability of Colloidal Gold Rapid Test for Protocol Definition

Currently, there is no gold standard for the identification of Covid-19 positive or negative subjects; however, the commonly adopted standard is the RT-PC, carried out with the methods suggested by the WHO [2]. We, therefore, selected the articles and data set, where the control was the RT-PCR, carried out on selected groups of people, certainly negative (e.g., healthy RT-PCR negative volunteers) or affected by Covid-19 (RT-PCR positive symptomatic patients).

The data for the test validation are derived from Li [10], Capello [30], Paradiso [31], Cassaniti [26] as well as the data provided by the manufacturer (provided by the producer Alpha Pharma Inc., Bitonto Bari 70031, Italy available on https://www.a-ps.it/). In the article by Paradiso [31], it was not possible to deduce the data referring only to IgM.

It should be noted that the specificity was very high in all studies (Table 1): All the trials included patients enrolled in the early stage of pandemics, with no known exposure to SARS-CoV-2 and no symptoms of Covid-19 (i.e., healthy volunteers with no contact and asymptomatic and with negative RT-PCR); those patients could be reasonably considered true negative subjects. Starting from this assumption, basically, no false positives were recorded. Nevertheless, because of the peculiarity of this infection, and because of the epidemiology of the pandemic, a negative RT-PCR and a negative medical history cannot rule out a possible SARS-CoV-2 infection even in asymptomatic patients, so that it would be difficult to compare the results of any test with a gold standard, aimed to assess the sensitivity and sensibility of the test itself. Thus, research protocols in the future should consider some specific features of the infection, of the way it spreads among the population, and of the immune response of the host to the disease. For this reason, the comparison with RT-PCR alone is not enough to establish the effectiveness of quantitative or qualitative serologic tests. Besides, the long-time persistence of immunoglobulins in the serum of the immunized persons should be addressed, as well as the absence of viral RNA at the sites of the swabs that can give negative PCR results in healed immunized patients. Table 1 shows the sensitivity and specificity data of the colloidal gold rapid test, versus RT-PCR oropharyngeal swab, as published in individual articles (Cassaniti-a column was computed based on data reported in the article relative to IgM).

Still, because of these reasons, the collected sensitivity values may vary widely among the different studies. Consequently, at present, we cannot establish a mean sensitivity value. In addition, the variations are possibly determined by the time of testing and by the eligibility criteria, which were not uniform in the different studies.

In any case, the high precision indicates the absence of analytical errors determined by cross-reactivity, as also reported by some authors [26].

**Figure 1:**
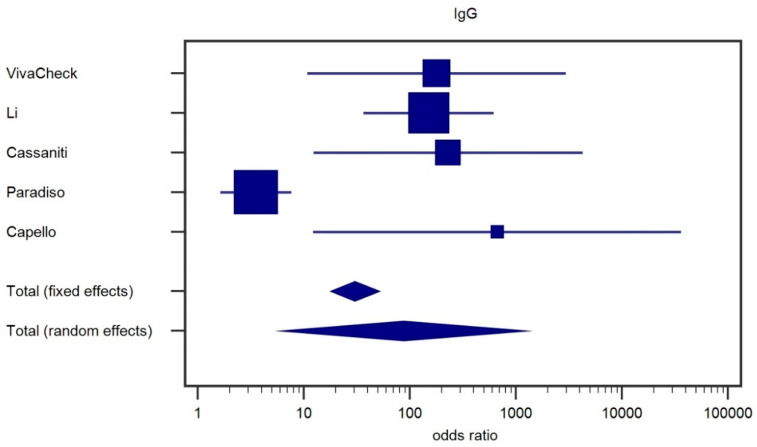
Forest plot for IgG.

**Figure 2:**
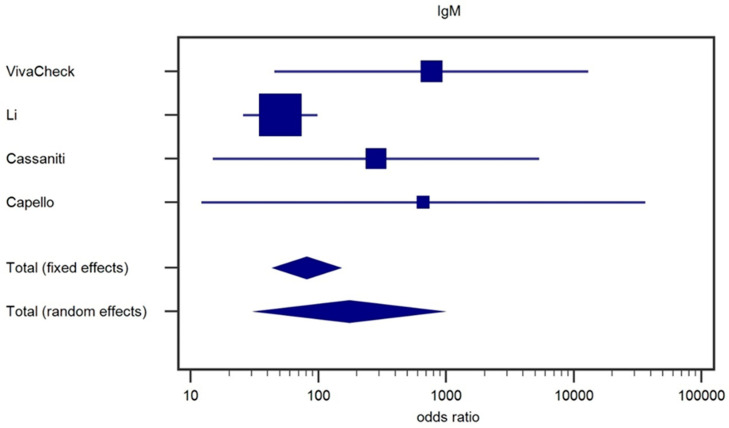
Forest plot for IgM.

To better understand the variability in sensitivity and better assess the accuracy of the test, we considered results of IgG and IgM testing separately. We performed the analysis via the meta analytic approach to evaluate the heterogeneity of the effect size; the results are shown in the forest plots of Figure 1 (IgG) and Figure 2 (IgM).

For the IgG (Figure 1) odds ratios, we found both significant fixed (*p* < 0.001) and random effects (*p* = 0.001); heterogeneity was very high, with Cochran’s Q = 4.61 (*p* < 0.0001), while the I2 statistic (accounting for variations due to actual heterogeneity and not to chance) was 90.2% (CI 95%: From 79.9% to 95.2%). Performing a second meta-analysis (data not shown) using all the pooled Cassaniti IgG data, we did not find substantial differences: For fixed effects we found again *p* < 0.001, whereas for random effects we obtained *p* = 0.002; Cochran’s Q was 37.78 (*p* < 0.0001), and the I2 statistic was 89.4% (CI95%: From 78.1% to 94.9%).

In addition, for the IgM (Figure 2) odds ratio, we found significant fixed and random effects (*p* < 0.001 in both cases); heterogeneity was less high than IgG; for Cochran’s Q we got 6.09 (*p* = 0.1072), thus a not significant heterogeneity, even keeping *p* < 0.1, as significance cutoff as suggested by Higgins et al., given the low number of studies [32] and the I2 statistic was found 50.8% (CI95%: From 0.0% to 83.7%). Furthermore, for IgM we performed a second meta-analysis (data not shown) using all the pooled Cassaniti IgM data: For fixed and random effects we found again *p* < 0.001, with a Cochran’s Q = 5.02 (*p* = 0.1702), and the I2 statistic was 40.3% (CI95%: From 0.0% to 79.8).

Both meta-analyses revealed a high inconsistency, at least in its broad confidence interval: The I2 statistic is given by the ratio (Q-D)/Q, where D accounts for the degrees of freedom of the system, e.g., the number of meta-analyzed studies minus one (in the case D > Q, then one keeps I2 = 0, with a CI of 95% from 0.0% to 100%). For this reason, adding a study to the meta-analysis can potentially decrease the inconsistency if the studies are very few, as in our case. However, the heterogeneity observed, and, therefore, the inconsistency, can also be explained by the differences in patient selection, and in the timing of the test (the time-lapse from time 0–the moment of the possible contact and infection–to the time the test was performed). It would be, therefore, advisable to produce clinical protocols that could standardize the procedure and the classification of patients, including the time of possible exposure, the prevalence of the disease in a population, the time of the onset of the symptoms, and the type and timing of other tests performed on the subjects. In fact, as the heterogeneity in sensitivity observed in the previous studies may depend on the different settings and the different timing, it would be advisable to serially test patients, recording and evaluating the immune response dynamics over time.

## 4. The Methodological Approach for the Design of a Research Protocol to Investigate COVID-19

Because of the complexity of this disease—whose fine clinical, immunological, and epidemiological features are mostly unknown and still under investigations—we cannot rely on the results of a test alone to make a diagnosis or to forecast the clinical evolution of the disease in a single patient as well as its epidemiological dynamics in a community. In the common practice, when we investigate a hypothesis, the trial we designed is aimed at assessing the main outcome of a single intervention or an exposure factor in a specific population. It means that in a research protocol, we consider one variable at a time, or in the better cases, a basic pool of parameters at a time. It also implies that we already know most of the big picture, so that we can concentrate on these fine details that may lead to different outcomes.

When it comes to COVID-19, however, we have to concede that we are in completely unexplored territory, with most of the clinical and epidemiological long-term implications impossible to evaluate or predict at the moment.

The scientific community is joining forces to fight the virus, but every group of study focuses only on specific aspects of the disease. A lot of pieces are, therefore, missing because, in such a brief time, it was almost impossible to portray the complexity of the whole picture. The amount of data is still limited, as well as the research settings and the experimental scenarios. In addition, most of the publications on this subject have been, reasonably, fast-tracked, skipping, or bypassing the traditional processes a research paper normally goes through. Some of these published papers are reports posted as letters to the editor, often reporting findings that—although accurate—represent a limited number of observations in specific groups and in specific conditions.

On the contrary, to better understand the virus, and to find useful hints on how to fight the disease, we must consider the whole picture and how every and each individual factor influence the results of a study.

In Table 2, we summarize some of the independent variables that should be theoretically addressed in each study, for a proper interpretation of any experimental observation or the evaluation of any outcome of an intervention.

Thus, if we want to understand the efficacy of a test, we have to integrate its result and the outcomes of any other investigation with other relevant information, as the clinical presentation (signs, symptoms, medical examination, laboratory investigation, imaging), the epidemiological background, and a patient’s medical history (to name a few: the time of the possible contact; epidemiological context; behaviors, actions or events that may have increased the chances of exposure; assessment of the risk profile of the patient, secondary to personal and professional conditions that could increase the chance of exposure; assessment of the risk of the possible exposure, secondary to the circumstances of the possible contact with other positive cases).

Besides, because we do not know how this virus behaves in the human body, we still must consider that there may be some clinical features, some analytes or other some peculiarities that come from other investigations that may be pathognomonic of the disease.

Table 3 summarizes typical symptom-described to date, imaging, and laboratory findings, which can suggest the diagnosis and prognosis of COVID-19. Although not specific, fever, dyspnea, and cough are common presentations of the disease, as well as a lymphocytopenia and increased lactate dehydrogenase. The role of Interleukin 38 and Interleukin 39 as a suppressor of inflammation has been recently studied [33], suggesting—as already observed with other immunomodulators—that the immune response of the host may be the cause of most of the complication of the disease [34]. Even if not of particular interest for research purposes, the modification of some clinical scores used to assess patients’ conditions has also been used to corroborate the diagnosis and the possible outcome of the infection.

The bottom line is that we must consider SARS-CoV-2 as an incognita. We do not know yet, and because this is a new infection, we will not know for a long period, if the virus can perpetuate in the human organism, if it can cause chronic sequelae, if it can remain dormant for decades and then relapses, if and how it can re-infected and cause a secondary disease, or if other microbiological factors, as well as other genetic or external factors, can affect the virulence of the virus in seropositive patients. Then, when researching COVID-19, we should record as many data as possible. It means that we must collect even the information that may not have a practical use at the moment, or that may appear irrelevant for the purpose of the study.

Therefore, we cannot produce research protocols only based on the evaluation of the clinical performance of a test, especially if the same test is aimed to screen patients or a population for diagnosis or follow up. On the contrary, we must gather all the possible available information to stratify the population better and understand how the test works in different scenarios. The level of uncertainty rises if we consider that because of the unicity of this virus, most of the time, we cannot establish when and how a patient or a population has been exposed to the same virus.

We, in fact, have to address the complexity, and we must become aware that we cannot rely on a simplified model to produce usable results.

**Figure 3:**
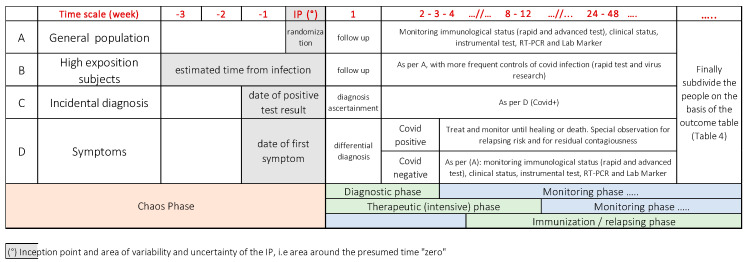
The timeline representing the different phases of the diseases. The interpretation of the results of the rapid tests depends on where the patients are in the timeline. The different groups are classified according to anamnestic criteria. The inception point varies fittingly. Each group represents different settings, where the rapid test can be used for different purposes: (**A**) Screening of a whole population for epidemiological reasons; (**B**) screening of a high-risk population; (**C**) screening in close contacts for diagnosis, follow-up and case tracking; (**D**) diagnosis and follow-up in symptomatic patients. In all case scenarios, the rapid test can provide information related to the possible acquired immunity to coronavirus. Finally, people and patients in each group should be divided according to the different outcomes shown in Table 4.

From this point of view, a major issue, scarcely addressed in most of the research papers in this field produced up to now, remains the inceptions’ point. In Figure 3, the specific timeline is presented.

Because the interpretation of the test rests on the inception point (time = 0; https://www.researchgate.net/publication/24398055_How_to_Design_a_Good_Case_Series and https://books.google.it/books?id=UWZNDwAAQBAJ&pg=PA303&lpg=PA303&dq=%22inception+point%22+clinical+methodology&source=bl&ots=lx6SU2yXYd&sig=ACfU3U2wKkmAKHKy0xH-zuus4NITLn1LYg&hl=it&sa=X&ved=2ahUKEwjFqqOmqIvpAhWB_aQKHYHHBf4Q6AEwDXoECAkQAQ#v=onepage&q=%22inception%20point%22%20clinical%20methodology&f=false) considered, we have to select a particular lapse time depending on the category the patients tested for Covid-19 belong to:

1. People with possible exposure to the virus (based on epidemiological context and/or on medical history). These people belong mainly to the general population or a specific group;
2. High-risk groups (healthcare workers, ridders) that may have an increased risk of exposition to a significant viral load;
3. People who incidentally underwent the test for several reasons, not related to clinical reasons;
4. Patients who developed mild to severe symptoms of Covid-19.

According to the risk of exposure, for each category, there is a pre-test positive probability difference that should be considered before the administration of the test.

Based on these argumentations, and to better understand the clinical and epidemiological potentiality of serological testing, we hereby present a template that we strongly suggest to use for the design of any study on diagnostic tools, and broadly on every research protocol aimed to investigate COVID-19.

### Crafting a Research Protocol for a Diagnostic Rapid Test

This is a research protocol based on a template addressing complexity, that is aimed mainly to investigate the possible use of a rapid test for the evaluation of the disease in a population when specific conditions are considered.

Although the result of a rapid diagnostic test offers only binary information (positive or negative IgM; positive or negative IgG), the result can be crucial in assessing a patient when all the complexity of this disease is considered. Moreover, it can provide a useful tool for the continuous screening of high-risk populations for the implementation of public health programs.

However, as advised before, the information coming from the test alone can be worthless if we do not consider the whole picture.

According to these criteria, we classified the patients to be tested according to the possible inception point, and, therefore, the possible clinical scenario. We then produced different groups, based on exposure and risk of exposure, on the selection criteria, and on the immune response to the different tests used to detect the infection. These criteria are summarized in Table 5.

This classification allows the evaluation of the upper and the lower limits of the time-lapse where we can place the inception point for each group considered, as for Figure 3.

Aside from selected and limited cases, in fact, we cannot establish the exact time of the infection. Yet, we can still gather valuable information if we evaluate the results of the test in different clusters and populations. We consider for this specific purpose patients routinely attending primary care facilities or an outpatient department, a general practitioner’s survey, or any other health center in the community.We might apply a similar scheme also in acute settings. However, we consider the peculiarity of the territorial emergency, where asymptomatic patients can unconsciously spread the infection, and the characteristics of a primary care department where patients can be followed-up over a longer period.

For our purpose, enrolled patients would be classified according to the groups presented in Table 5, and for their demographic features. In addition, all the available information on the recent and past medical history should be recorded, with a particular focus on pre- and post-COVID-19 results of laboratory testing and other investigations (see Table 3), and on behaviors or medical conditions that may increase the risk of becoming infected and of becoming seriously ill.

Patients will be serially tested with the colloidal gold VivaDiag™ COVID-19 IgM/IgG Rapid Test. The suggested frequency is weekly. A shorter interval may be suggested in high-risk settings. Each patient enrolled in the study would perform an RT-PCR test at the beginning of the study and if symptomatic.

Patients should be regularly tested up to six months after the estimated time of contagion. Investigators will record the symptoms and any other medical investigation together with their date of onset and resolution.

The primary outcomes are:

1. To establish the accordance of the test with the clinical features of the disease and to assess how the test results change over time in the different clusters of patients, as summarized in Table 5.
2. To establish the reliability of the serologic rapid test to detect a SARS-CoV-2 infection and, therefore, its sensitivity and specificity when the integration of anamnestic, clinical, laboratory, molecular, imaging criteria are considered as the gold standard.

The secondary outcome is the comparison of the serological rapid test with the RT-PCR commonly used to make an etiological diagnosis of Covid-19.

In fact, we cannot classify patients according to the results of a single test is positive or negative patients. Moreover, the immune response of the host is still unknown, as well as the level of immunity secondary to the infection. Thus, the negative or positive status cannot be assessed and cannot be established by the result of a test. The serological response should be interpreted with information coming from the clinical reports. Consequently, before drafting conclusions, the data analysis at the end of the study must consider the following considerations:

- If the test is positive, even in the presence of a negative PCR, the patient should be considered positive and treated according to his or her clinical picture. When IgM alone is positive, the serological test shows a recent infection, even if the patient has not developed any symptoms yet. On the other hand, if the test is positive for IgG alone and the patient is asymptomatic, the result can be used for epidemiological purposes. Some recent investigations [27], however, show an inconsistent response of IgM and IgG in different patients, with a seroconversion of both the immunoglobulin classes that reach 100% after a window period.
- If the test is negative in a symptomatic patient with a high exposure risk, the serological test should be routinely repeated, considering the window period between the time 0 of the infection and the production of circulating antibodies.

Even if rapid tests are designed to reduce the measure errors, to avoid a false negative, it is essential to follow the producer’s instructions to avoid procedural mistakes. For this reason, we chose for our research protocol a test based on a very easy procedure, such as those already in use for other diseases also in very limited settings and laboratories with very poor resources, or auto-diagnostic kits. The test is also easy to perform and to read for minimally trained personnel. Nevertheless, because we do not have yes-or-no tests available and given the characteristic of Covid-19, it is unlikely that those can be created in the future, the clinical assessment of the patients remains crucial. This implies that medical history should be correctly collected, and clinical signs must be accurately recorded to increase the chance of making a proper diagnosis, prognosis, and management plan.

Lastly, all the data collected should be shared with other researchers to prompt further studies, and, consequently, the whole dataset should always be made available.

This protocol dubs a first research trial (ClinicalTrials.gov Identifier: NCT04316728. Available from http://clinicaltrials.gov/ct2/show/NCT04316728) aimed at evaluating the immune response of negative patients during a COVID-19 outbreak, plus the clinical performance of the test in early detection the infection, and the reliability of the test in those patients who develop clinical signs of COVID-19 during the trial. Preliminary unpublished data match the observations reported in Table 1, showing a good correspondence with other research studies and also suggesting a role of serological test for the screening of asymptomatic patients.

## 5. Conclusions

The 2020 novel coronavirus pandemic is showing how weak the idea of global health is. This worldwide emergency asks and needs prompt answers and original solutions. The early diagnosis of coronavirus remains a major issue, as a proper screening strategy can improve the design and implementation of workable public health models aimed to confine the virus and to reduce its devastating effect on the population. It is, therefore, paramount—also considering the clinical complexity of this new disease—to develop accurate, user-friendly kits, that can help doctors and healthcare workers to make a diagnosis of Covid-19. Thus, molecular scientists must be front-runners in this race against the virus, providing their expertise and the unique research capability of their field.

We investigated the quick detection approach targeting viral IgM or IgG antibodies, with the colloidal gold-based immunochromatographic (ICG) strip assay, in comparison with real-time RT-PCR testing. The advantages of the ICG strip viral antibody detection are obvious. First of all, the sensitivity of the ICG strip is comparable, especially after 7 days of onset; the detection capacity in nuclear acid-negative “clinically diagnostic” patients is impressive. Therefore, ICG strips are highly recommended to be utilized as the supplementary diagnostic approach in clinical applications. The ICG strip can also be widely adopted in areas where diagnostic capacity is limited. Secondary, the ICG strip assay is operational as it is ready to use and timesaving. The assay can be finished within 15 min without specialized equipment. Third, unlike oral swab sampling that may cause stimulated retching and coughing, which increases the exposure risk of laboratory technicians, the blood collection could avoid the unnecessary risk and reduce the operation steps that may cause aerosol. Fourth, the detection of antibodies may also indicate the disease recovery, as immunoglobulins are among the most important soldiers in the battle of viruses. Patients who initially detected as virus-positive and gradually became negative but with detectable IgG or IgM during the disease progression may be considered as recovered from this battle. It is noteworthy that the ICG strip can only provide qualitative results, but the serological ELISA assay against the viral antibody offered the quantitative antibody titer and is regarded as the superior alternation at this point. Last, but not least, the strip can be used for community surveillance and, therefore, for the design of public health strategies aimed to confine the dissemination of the virus, to contain an ongoing epidemic, and to call off whatever restrictions and measures are needed to reduce the spread of the disease in a timely manner.

In conclusion, the main advantages of the serological test are higher accuracy in detecting a SARS-CoV-2 infection even in asymptomatic patients or before the onset of the symptoms when the test is performed after a significant amount of time from the suspect day of contagion, and the simpler way of use that allows operators with minimal training to screen a high number of people with fewer sampling mistakes. Finally, it reduces the exposure of health workers performing the test to the virus.

Taking into account the background and, in particular, long results [27], we speculate that serological tests, although needing to be more thoroughly studied, could be indispensable weapons for front-line doctors who work in a decentralized setting to have rapid answers.

Therefore, it is crucial to gather all the possible information every time a patient is tested and assessed to understand better how the infection and the immune response work so that we can build risk classes and individual and population profiles that could help clinicians and health policy-makers to define a better strategy to fight the infection and the spread of the disease, allowing at the same time, people and society to safely go back to normal or more regular life.

We finally endorse and foster a proactive collaboration among molecular researchers and clinicians, with the creation of multicentric studies aimed to produce tools that can be used as weapons for the war against this invisible enemy that may undermine the future of the same humankind.

## Figures and Tables

**Table 1:** Colloidal gold rapid test sensitivity and specificity versus real-time polymerase chain reaction (RT-PCR) in different experimental settings (VivaChek, Cassaniti-a, Capello: Volunteers, healthcare workers, and patients; Li, Cassaniti-b, Paradiso: Patients tested in specific settings).

	VivaCheck	Li	Cassaniti-a (*)	Cassaniti-B	Paradiso	Capello
Test (*n*)	350	397	60	50	191	26
Sensitivity	81.2 ^(^)^	88.6	83.3	18.4	30.0	100.0
Specificity	100.0	90.6	100.0	9.7	89.0	100.0

^(^)^ between 4–10 days; 97.1% > 10th (*) estimated on the basis of published data.

**Table 2:** Prototypic examples of independent variables that should be considered when clustering the results and evaluating the outcomes. For a well-known disease, some of these variables are known. Therefore, we cannot estimate a priori whether they can or cannot affect the outcome and design the research protocol, accordingly, setting inclusion or exclusion criteria. For COVID-19, the weight of every variable in affecting the outcome of an experimental observation or intervention is generally unknown.

Type of Variable	Possible Independent Variable
Demographic and environmental feature	Age
	Sex
	Ethnicity
	Occupation
	Geographical location and Climatic factors, Pollution
	Socio-economic status
Physiological Features	Diet
	Level of physical activity
	Immunization Status
	Genetic Subtype
Comorbidity	Health patients
	Concomitant acute condition
	Chronic conditions and frailties
	Smoke and other risk factors
Viral Physiology	Viral strain
	Tropism
	Elusion mechanisms of virus
Physio-pathology of the disease	Virulence
	Immune response of the host
Clinical assessment and investigations	Symptoms (*)
	Clinical signs (*)
	Laboratory findings (*)
	Instrumental investigation (*)
	Analytes Considered (*)
	Drugs

The star (*) indicates some parameters that can be considered as covariates or as dependent variables, depending on the studies.

**Table 3:** Clinical, laboratory, and imaging findings commonly found in patients with SARS-CoV-2 infection.

Clinical and Laboratory
**Symptoms**
Fever (temperature ≥ 37.3 °C); cough, sputum, shortness of breath, myalgia, fatigue, diarrhea, nausea and vomiting, conjunctivitis, anosmia, dysgeusia…
**Comorbidities**
Hypertension, heart failure, coronary heart disease, diabetes, kidney failure, cancer, chronic obstructive lung disease, immunodeficiency, stroke, cerebrovascular accident, gastrointestinal disease, transplant…
**Laboratory findings**
White blood cell count, lymphocyte count, hemoglobin, platelet count, albumin, creatinine, ALT/AST, lactate dehydrogenase, high-sensitivity cardiac troponin I, prothrombin time, D-dimer, IL-6, IL-1, IL-8, IL 38; IL-39, TNFα, CCl-2, CCL-3, CCL-5, IP-10, MCP-1, procalcitonin, C-reactive protein, pH, lactate, vitamin D
**Score**
Curb-65; quick-SOFA, SOFA, APACHE II
**Imagine findings**
Consolidation, ground-glass opacity, bilateral pulmonary infiltration
**Histo- and cytopathological findings**
Sign of inflammation, alveolar damage with exudate, lymphocyte, multinucleated giant cells…

SOFA = sequential organ failure assessment. qSOFA = quick SOFA. ALT = alanine aminotransferase. AST = aspartate aminotransferase; IL = interleukin, TNFα = tumor necrosis factor. CCL = C-C motif chemokine ligand. IP-10 = IFNγ-induced protein 10; MCP-1 = monocyte chemoattractant protein 1.

**Table 4:** The table summarizes the main possible outcomes of Covid-19. It suggests what measures will be needed in the long-term, defining what protocols are needed in designing a study tailored to given categories of people. Using the correct protocol, in fact, is the weapon to fight the infection. Indeed, a detailed and clear protocol should be proposed to study and stratify patients’ outcomes. The table should be read as an extension of Figure 3 to define a patient’s history.

Outcomes	Clinicians/Epidemiologists	Research/Protocols (Examples)	Notes
Death	Clinical observation and autopsy	Nested case-control, pathophysiological approach	Monitoring of all parameters, including in-deep laboratory investigations, PCR, microscopy, to define the initial cause of death, the final one and contributing cause
Relapse of the disease	In-deep permanent clinical observation	Ecological study, clinical reports, case control	Study immune response and virus variability
Chronicity of the disease	Clinical observation	Cross-sectional survey, clinical reports, case control	Evaluate comorbidities, aging, chronic drug intake
Relapse of the disease in a healed patient	In-deep permanent clinical observation	Epidemiological surveys, case controls, nested case control	Study immune response and virus variability
Patient Healed, immunized	Follow-up	Epidemiological surveys, cohort study	Patients need to be studied in the long-time to avoid unexpected relapse
Non infected, healthy	Special follow-up, particularly in exposed/working subjects	Ecological study, epidemiological surveys	Prevent the infection with appropriate measures of disease control, waiting for the vaccine. At least two serological tests should be administered.
Not infected, healthy, elderly or high-risk subject	In-deep follow-up	Ecological study, longitudinal cohort study	Prevent the infection, studying the major comorbidities which can modify the prognosis.

**Table 5:** The different classification of the patients enrolled in the study. The different clusters represent different clinical and epidemiological scenarios. The information coming from the clinical history of a patient combined with the results of the test and its modification over time will serve to portray a picture of the possible interpretation of the test according to the different phases, as per Figure 3 timeline.

Group Classification	Main Clusters
**Exposure/risk classification**	People with known close contact People with no clear history of close contacts People constantly exposed
**Selection Criteria**	Randomized population High exposition subjects Incidental diagnosis (see text) Symptomatic patient’s data
**Diagnostic criterium**	Clinical criteria Serological testing Molecular testing Pathognomonic laboratory findings Pathognomonic instrumental findings
